# Control of Interface Defects for Efficient and Stable Quasi‐2D Perovskite Light‐Emitting Diodes Using Nickel Oxide Hole Injection Layer

**DOI:** 10.1002/advs.201801350

**Published:** 2018-10-04

**Authors:** Seungjin Lee, Da Bin Kim, Iain Hamilton, Matyas Daboczi, Yun Seok Nam, Bo Ram Lee, Baodan Zhao, Chung Hyeon Jang, Richard H. Friend, Ji‐Seon Kim, Myoung Hoon Song

**Affiliations:** ^1^ School of Materials Science and Engineering and Low Dimensional Carbon Center and KIST‐UNIST Ulsan Center for Convergent Materials Ulsan National Institute of Science and Technology (UNIST) UNIST‐gil 50 Ulsan 44919 Republic of Korea; ^2^ Department of Physics and Centre for Plastic Electronics Imperial College London Prince Consort Road London SW7 2AZ UK; ^3^ Department of Physics Pukyong National University 45 Yongso‐ro, Nam‐Gu Busan 48513 Republic of Korea; ^4^ Cavendish Laboratory JJ Thomson Avenue Cambridge CB3 0HE UK

**Keywords:** defects, nickel oxide, perovskite light‐emitting diodes, stability

## Abstract

Metal halide perovskites (MHPs) have emerged as promising materials for light‐emitting diodes owing to their narrow emission spectrum and wide range of color tunability. However, the low exciton binding energy in MHPs leads to a competition between the trap‐mediated nonradiative recombination and the bimolecular radiative recombination. Here, efficient and stable green emissive perovskite light‐emitting diodes (PeLEDs) with an external quantum efficiency of 14.6% are demonstrated through compositional, dimensional, and interfacial modulations of MHPs. The interfacial energetics and optoelectronic properties of the perovskite layer grown on a nickel oxide (NiO*_x_*) and poly(3,4‐ethylenedioxythiophene):polystyrene sulfonate hole injection interfaces are investigated. The better interface formed between the NiO*_x_*/perovskite layers in terms of lower density of traps/defects, as well as more balanced charge carriers in the perovskite layer leading to high recombination yield of carriers are the main reasons for significantly improved device efficiency, photostability of perovskite, and operational stability of PeLEDs.

Metal halide perovskites (MHPs) have emerged as a promising class of materials for optically pumped lasers and light‐emitting diodes (LEDs) owing to their narrow emission spectrum and wide range of color tunability.^1^, ^2^, ^3^, ^4^, ^5^, ^6^, ^7^ To date, perovskite LEDs (PeLEDs) have recorded an external quantum efficiency (EQE) of up to 14.36% for green emissions^6^ and 12.7% for near‐infrared emissions.^7^ However, the low exciton binding energy in MHPs is a fundamental limitation that leads to the generation of free charges at room temperature. The competition between the trap‐mediated nonradiative recombination and the electron–hole radiative bimolecular recombination causes a low photoluminescence quantum yield (PLQY) and a PLQY dependence on the excitation intensity.^8^, ^9^ Therefore, low trap density in MHPs is required to increase radiative bimolecular recombination rate for high PLQY and EQE values of PeLEDs.

The choice of monovalent organic cation can strongly influence the optical and electrical properties of MHPs.^10^, ^11^, ^12^, ^13^, ^14^ Many researchers have reported that formamidinium lead halide shows remarkably enhanced carrier diffusion length compared with methylammonium lead halide.^10^, ^11^, ^12^ Hanusch et al. showed that the carrier lifetime in polycrystalline films of formamidinium lead bromide (FAPbBr_3_) is much longer than that of methylammonium lead bromide (MAPbBr_3_).^10^ Moreover, Zhumekenov et al. reported that FAPbBr_3_ single crystals show superior optical and electrical properties, with a much longer carrier diffusion length and a lower dark current, to MAPbBr_3_,^11^ which they attributed to the considerably lower trap density of FAPbBr_3_. Thus, FAPbBr_3_ is a potential candidate for light‐emitting material for high‐performance PeLEDs.

In LED applications, the charge balance injected from charge transport layers (CTLs) is crucial for maximizing the recombination rate in the emitting layer for high LED performance. Ideal CTLs need to meet suitable energy levels for the injection of charges and efficient charge transport by blocking opposite charges.^15^, ^16^ The bottom CTL also influences the growth of the perovskite crystal and its quality of interface,^17^, ^18^, ^19^, ^20^, ^21^ which is very important for the performance of PeLEDs. Defects in the interface of perovskite form deep‐level traps inside the bandgap,^20^, ^21^ which can be the dominant nonradiative recombination channel. For the organic hole transport layer (HTL), poly(3,4‐ethylenedioxythiophene):polystyrene sulfonate (PEDOT:PSS) is commonly used as a HTL. However, there is evidence that its acidic and hydroscopic nature severely deteriorates long‐term stability.^22^, ^23^, ^24^ Moreover, PEDOT:PSS causes the quenching of luminescence at its interface with the perovskite layer, which degrades device performance. As an alternative to PEDOT:PSS, such stable inorganic p‐type materials as copper thiocyanate (CuSCN), vanadium oxide (V_2_O_5_), molybdenum oxide (MoO_3_), and nickel oxide (NiO*_x_*) have been introduced as promising HTLs.^25^, ^26^, ^27^, ^28^, ^29^, ^30^, ^31^, ^32^, ^33^ These metal oxides have the advantages of good air stability, high transparency, and high carrier mobility. Furthermore, crystalline metal oxides can facilitate the growth of highly crystalline perovskite and improve the quality of interface between metal oxides and perovskite,^34^, ^35^ leading to a reduced path for nonradiative recombination.

In this study, we control the trap density of perovskite through compositional, dimensional, and interfacial modulations, which substantially increase bimolecular radiative recombination by outcompeting trap‐mediated nonradiative recombination. Owing to the low trap density, long‐living free carriers of formamidinium (FA) based perovskite are allowed to recombine in small radiative domains through efficient and fast energy transfer by modulating the dimensionality. Moreover, we investigate interfacial defects of perovskite deposited on NiO*_x_* and PEDOT:PSS through ambient pressure air photoemission spectroscopy (APS), the Kelvin probe (KP), and surface photovoltage (SPV) for perovskite films of varying thicknesses. Crystalline NiO*_x_* enables the growth of highly crystalline perovskite with fewer interface defects, which enhances the optical properties and photostability of perovskite as well as the operational stability of PeLEDs. Through effective control over the trap density of perovskite, we demonstrate efficient and stable green emissive PeLEDs with a maximum luminance of 24 100 cd m^−2^, maximum current efficiency (CE) of 62.4 cd A^−1^, and maximum EQE of 14.6%.

To investigate the optical properties and thermal stability of perovskites using FA and methylammonium (MA), we obtained FAPbBr_3_ and MAPbBr_3_ films with similar morphologies and grain sizes using the same fabrication method and condition (Figure S1, Supporting Information). Despite their similar morphologies, the FAPbBr_3_ and MAPbBr_3_ films showed significantly different properties. Although the reason for the remarkable difference in the properties of perovskites using FA and MA is unclear, previous reports have proposed that the outstanding properties of FA‐based perovskite can be attributed to its low trap density.^10^, ^11^ Time‐resolved and steady‐state photoluminescence (PL) spectra were measured to investigate the optical properties of FAPbBr_3_ and MAPbBr_3_ (**Figure** **1**a,b). The PL lifetime of FAPbBr_3_ was significantly greater than that of MAPbBr_3_, indicating that free carriers in FAPbBr_3_ were able to survive for a long time without being trapped. The long diffusion of the free carriers increased the probability of bimolecular recombination to regain their bound forms for radiative decay. Consequently, FAPbBr_3_ showed a higher PL intensity than MAPbBr_3_ (Figure 1b). FAPbBr_3_ exhibited red‐shifted band edge absorption and PL emission owing to a smaller bandgap than MAPbBr_3_ (Figure S2, Supporting Information), which can be attributed to a larger lattice constant of FAPbBr_3_ on account of the large size of FA. To examine the thermal stability of the perovskite depending on the monovalent organic cation, X‐ray diffraction (XRD) patterns and photographs of perovskite films were observed before and after thermal annealing (Figure 1c–e). The decomposition process can be traced through the disappearance of the (100) peak in the XRD patterns of perovskites and the change in film color. The (100) peak in XRD patterns of MAPbBr_3_ disappeared, and the MAPbBr_3_ film turned into a colorless and transparent film after annealing at 150 °C for 2 h, which indicated that the MAPbBr_3_ film had completely decomposed. By contrast, the FAPbBr_3_ film exhibited relatively good thermal stability, showing still the (100) XRD peak and orange color even after annealing at 175 °C for 2 h. However, the FAPbBr_3_ film was completely decomposed after annealing at 200 °C for 2 h, showing no (100) XRD peak or colorless film. The thermal stability of FAPbBr_3_ can be attributed to the fact that the FA cation has a higher probability of forming a hydrogen bond with the inorganic frame than the MA cation.^36^, ^37^

**Figure 1:**
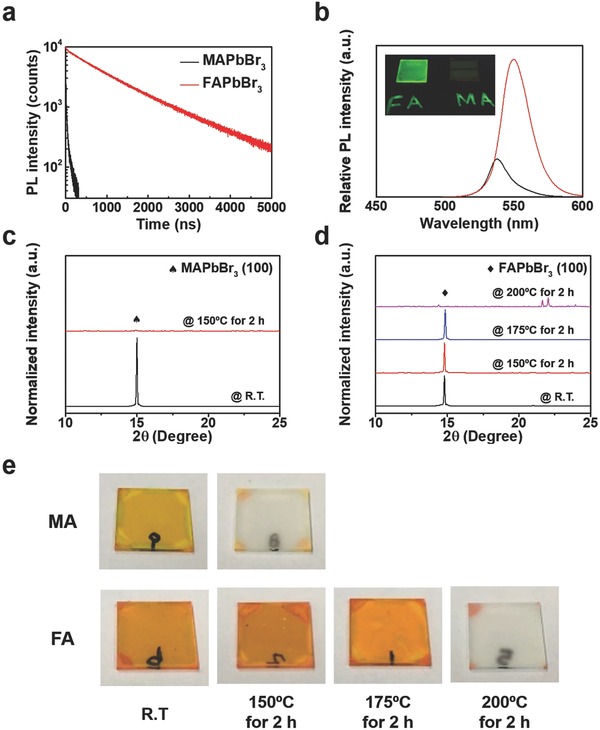
Optical properties and thermal stability of MAPbBr_3_ and FAPbBr_3_. a) Time‐resolved PL spectra, and b) steady‐state PL spectra of MAPbBr_3_ and FAPbBr_3_. Normalized XRD patterns of c) MAPbBr_3_ and d) FAPbBr_3_ before and after thermal annealing. e) Photographs of MAPbBr_3_ and FAPbBr_3_ before and after thermal annealing.

A schematic illustration of the device design and quasi‐2D perovskite (average *n* value = 3) along with the chemical structure of the benzylammonium (BA) incorporated as a bulky ammonium cation to fabricate the quasi‐2D perovskite is represented in **Figure** **2**a. The cross‐sectional scanning electron microscopy (SEM) image of PeLEDs with a multilayered structure of indium tin oxide (ITO)/NiO*_x_*/quasi‐2D perovskite (*n* = 3)/ 2,2′,2″‐(1,3,5‐benzinetriyl)‐*tris*(1‐phenyl‐1‐*H*‐benzimidazole) (TPBi)/LiF/Al was observed (Figure 2b). The dimensional modulation of quasi‐2D perovskites can be achieved by adjusting specific stoichiometric quantities of lead bromide (PbBr_2_), formamidinium bromide (FABr), and benzylammonium bromide (BABr). The structural evolution from 3D to quasi‐2D perovskite can be monitored by XRD, and absorption and emission spectra. The XRD patterns of 3D FAPbBr_3_ showed a peak at 14.80° corresponding to the (100) plane of the cubic phase, whereas the quasi‐2D perovskite with *n* = 2, 3, and 5 showed peaks at small angles (2θ < 10°) owing to their expanded unit cells (Figure 2c). The absorption spectrum of the 3D FAPbBr_3_ showed no absorption peaks associated with the large bandgap of quasi‐2D perovskites, whereas the absorption spectrum of the quasi‐2D perovskites clearly showed absorption peaks at high‐energy states of the quasi‐2D perovskites (Figure 2d). The quasi‐2D perovskite with lower average values of *n* exhibited stronger absorption peaks at higher energy states as blue‐shifted PL spectra owing to their lower dimensionality (Figure 2d,e). We consider the blueshift of PL to be enabled by recombination at higher energy states of quasi‐2D perovskite with smaller n values. Past studies on quasi‐2D perovskites have shown that photogenerated excitons are concentrated and confined in the smaller bandgap emitters through energy transfer, which increases the local charge density and rate of radiative bimolecular recombination. To confirm the improvement in the optical properties of the quasi‐2D perovskites, time‐resolved and steady‐state PL measurements were performed (Figure S3, Supporting Information). The steady‐state PL spectra showed that the PL intensities of the quasi‐2D perovskite were higher than those of 3D FAPbBr_3_. The quasi‐2D perovskite with *n* = 3 showed the highest PL intensity and a high PLQY (53.1%) compared with 3D FAPbBr_3_ (15.1%). It also yielded a substantially shorter PL lifetime (0.14 µs) than the 3D FAPbBr_3_ (4.66 µs; Table S1, Supporting Information), which indicates fast bimolecular recombination by effectively focusing energy on radiative domains.

**Figure 2:**
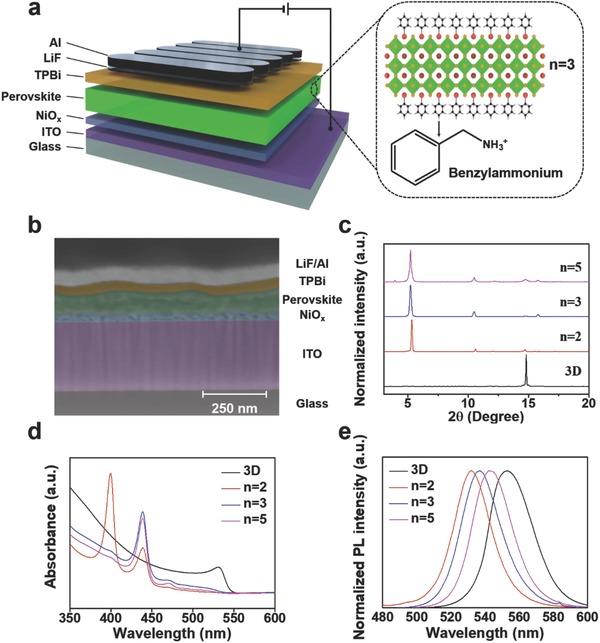
Schematic of device structure, chemical structure, cross‐sectional SEM image, XRD, absorption and PL. a) Schematic of the structures of PeLED and quasi‐2D perovskite (*n* = 3), and the chemical structure of BA. b) Cross‐sectional SEM image of the PeLED device. c) Normalized XRD patterns, d) absorbance, and e) normalized PL spectra of 3D FAPbBr_3_ and quasi‐2D perovskites with *n* = 2, 3, and 5.

To investigate defects in the interface of the perovskite deposited on NiO*_x_* and PEDOT:PSS, APS, KP, and SPV were executed on the perovskite films (at 15, 35, and 120 nm thicknesses) deposited on NiO*_x_* and PEDOT:PSS. The photoemission spectra of the perovskite films deposited on NiO*_x_* and PEDOT:PSS at thicknesses of 15 and 120 nm are shown in **Figure** **3**a,b. The different thicknesses of these films were expected to represent the interface and bulk‐like properties. For the 15 nm thick samples, the extrapolated highest occupied molecular orbital (HOMO) values of the perovskite were 5.35 ± 0.05 and 5.45 ± 0.05 eV deposited on NiO*_x_* and PEDOT:PSS, respectively, whereas those for the 120 nm thick samples increased to 5.50 ± 0.05 and 5.55 ± 0.05 eV, respectively. The reduced difference in HOMO values for the 120 nm thick films indicates that while the bulk energy levels of the perovskite remained similar, at the HTL/perovskite interface, the perovskite HOMO was strongly influenced by the HTL underneath. This is most likely caused by the local electronic densities of states of the perovskite altered at/near the HTL. This was further confirmed by the similarities of the APS spectra of the 120 nm thick film to those of the 35 nm thick film, which showed the same extrapolated HOMO values (5.50 ± 0.05 and 5.55 ± 0.05 for NiO*_x_* and PEDOT:PSS, respectively; Figure S4a, Supporting Information). All the extrapolated HOMO energy levels were shown in Figure 3c. From the measured HOMO values of the PEDOT:PSS (5.15 eV) and NiO*_x_* (5.18 eV), a relatively larger interfacial energetic barrier for the hole injection is calculated for PEDOT:PSS/perovskite than NiO*_x_*/perovskite samples (0.30 vs0.17 eV), implying that NiO*_x_* would show better hole injection properties when used in perovskite LEDs.

**Figure 3:**
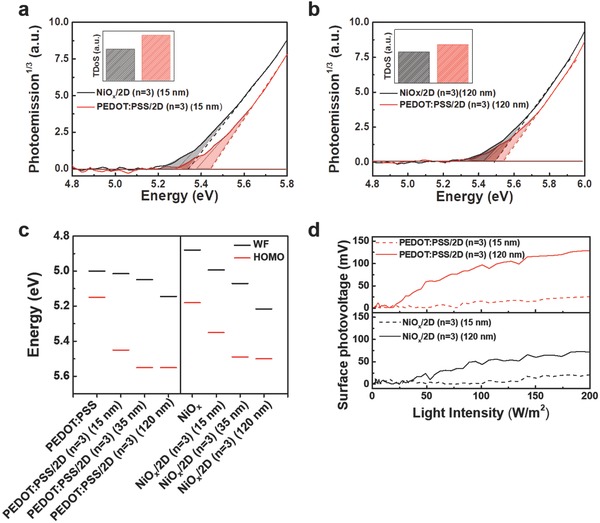
APS, WF, HOMO, and SPV measurements for quasi‐2D perovskite with *n* = 3 deposited on NiO*_x_* and PEDOT:PSS. APS spectra for a) 15 nm thick and b) 120 nm thick perovskite films deposited on NiO*_x_* and PEDOT:PSS. c) WFs and HOMO for pristine NiO*_x_* and PEDOT:PSS, as well as perovskite films of 15, 35, and 120 nm deposited on NiO*_x_* and PEDOT:PSS. d) SPV for 15 nm thick (dashed) and 120 nm thick (solid) perovskite films deposited on NiO*_x_* and PEDOT:PSS as a function of light intensity.

The threshold of energy for photoemission was below the extrapolated HOMO value, which can be attributed to the presence of the sub‐bandgap trap/defect states within the perovskite.^38^, ^39^ Thus, a relative comparison of trap states could be made by comparing the integrated area below the photoemission threshold (shaded area in Figure 3a,b). We found that 15 nm thick films of perovskite deposited on PEDOT:PSS showed a (≈1.45 times) larger area occupied by the subgap states (inset in Figure 3a) than those deposited on NiO*_x_*, indicating that the perovskite deposited on NiO*_x_* had lower trap density than that deposited on PEDOT:PSS. Interestingly, for the 35 and 120 nm thick films, the extracted subgap area of PEDOT:PSS was only ≈1.3 and ≈1.2 times greater than that of the NiO*_x_* samples, suggesting that relatively higher density of the trap states was preferentially formed near the interface of PEDOT:PSS and the perovskite.

The work function (WF) values were also measured for 15, 35, and 120 nm thick perovskite films deposited on PEDOT:PSS and NiO*_x_* and shown in Figure 3c. We observe that the Fermi levels for the 15, 35, and 120 nm thick perovskite films deposited on PEDOT:PSS (5.02, 5.05, and 5.15 eV, respectively) were strongly influenced by the WF of the PEDOT:PSS (measured at 5.00 eV), i.e., the Fermi level of the perovskite was “pulled” toward that of PEDOT:PSS.^40^, ^41^ This implies that the depletion width of the perovskites on PEDOT:PSS extended through the bulk of the film.^42^ On the contrary, perovskite films deposited on NiO*_x_* were slightly less influenced by the WF of the NiO*_x_* film (measured at 4.88 eV). The Fermi levels of the 15, 35, and 120 nm thick perovskite films deposited on NiO*_x_* were 5.00, 5.07, and 5.21 eV, respectively.

Trap states located on the surface of the semiconductors or the interface of two semiconductors can cause the accumulation of charges, which can develop band bending.^43^ Under illumination, SPV can be generated by the movement of an excessive number of photogenerated charge carriers to fill in these trap states, screening the internal electric field, and reducing band bending. Thus, the magnitude of the SPV can be related to the amount of density of the trap states.^43^, ^44^ The SPV values as a function of incident light intensity for 15 and 120 nm thick perovskite films deposited on NiO*_x_* and PEDOT:PSS were measured (Figure 3d). Once the light was turned on, the PEDOT:PSS samples showed an increase in SPV to ≈26 and 129 mV from the dark values of the 15 and 120 nm perovskite films, respectively. The 15 and 120 nm perovskite films deposited on NiO*_x_*, however, showed an increase only to ≈20 and 72 mV, respectively. The difference in SPV values between the thin and thick films were thus 103 mV for PEDOT:PSS and 52 mV for NiO*_x_* samples. A much larger change in SPV values for PEDOT:PSS samples might indicate a higher density of trap states present at the PEDOT:PSS samples compared with NiO*_x_* samples.

The crystalline NiO*_x_* film enables the growth of highly crystalline perovskite and reduces trap states at the interface of NiO*_x_* and the perovskite layer. The morphologies of the perovskite films deposited on NiO*_x_* and PEDOT:PSS were observed using SEM (Figure S5, Supporting Information). The surface of the perovskite film deposited on NiO*_x_* showed neat morphology without no other shapes of crystallites. In contrast, the surface of the perovskite film deposited on PEDOT:PSS was intricately covered with various shapes of crystallites, which may be attributed to the acidic characteristics of PEDOT:PSS. The morphologies of the perovskite films deposited on PEDOT:PSS with different pH values were compared to investigate the effect of their acidity on the growth of perovskite film. To adjust pH values of PEDOT:PSS, acidic PEDOT:PSS (AI 4083, Celvios) was titrated with imidazole. The pH values of acidic, neutral, and basic PEDOT:PSS were measured to be 1.95, 7.5, and 8.5 using a pH meter. The surface of the perovskite films deposited on pH neutral and basic PEDOT:PSS showed clean morphology without no crystallites, which indicates that the acidic characteristics of PEDOT:PSS have a negative effect on crystal growth. Moreover, steady‐state PL spectra and photographs showing PL emission of perovskite films deposited on NiO*_x_* and PEDOT:PSS with different pH values were observed to investigate the effect of their acidity on optical properties of perovskite (Figure S6, Supporting Information). Perovskite films deposited on pH neutral and basic PEDOT:PSS showed higher PL intensity than that deposited on acidic PEDOT:PSS, but they showed inferior PL intensities to perovskite film deposited on NiO*_x_*. The acidity of PEDOT:PSS have a negative effect on optical properties of the perovskite, and other factors such as hygroscopic and amorphous characteristics may lead to poor interface quality of the perovskite compared with NiO*_x_*. To further investigate the optical properties of the perovskite films deposited on NiO*_x_* and acidic PEDOT:PSS, their PL lifetimes, PL intensities, and PLQYs were measured (Figure S7a–c, Supporting Information). The perovskite deposited on NiO*_x_* showed a longer PL lifetime, and higher values PLQY compared with the perovskite deposited on PEDOT:PSS. This can be attributed to the fewer trap/defect states. Moreover, the stability of the perovskite deposited on NiO*_x_* and PEDOT:PSS in terms of PL was measured at constant laser excitation using confocal microscopy (Figures S7d and S8, Supporting Information). The confocal PL images of the perovskite deposited on NiO*_x_* exhibited bright and full coverage emission, whereas images of the perovskite deposited on PEDOT:PSS showed dark regions dominated by nonradiative recombination. Moreover, highly crystalline perovskite with fewer defects deposited on NiO*_x_* showed better photostability in terms of PL under laser excitation than that deposited on PEDOT:PSS.

The NiO*_x_* films were optimized by testing different concentrations of precursor NiO*_x_* solutions to balance the charge carriers through efficient hole transport and electron blocking (Figure S9 and Table S2, Supporting Information). To investigate improved hole injection properties of NiO*_x_*, We fabricated hole‐only devices (ITO/NiO*_x_* or PEDOT:PSS/BA_2_FA_2_Pb_3_Br_10_/TFB/MoO_3_/Au) and electron‐only device (ITO/ZnO/BA_2_FA_2_Pb_3_Br_10_/TPBi (60 nm)/LiF/Al; Figure S10, Supporting Information). The hole current density of hole‐only device fabricated with NiO*_x_* was higher than that with PEDOT:PSS, showing better hole injection properties when NiO*_x_* is used as HTL in perovskite LEDs. Moreover, hole current density of hole‐only device fabricated with NiO*_x_* was well matched with electron current density of electron‐only device at the condition of 60 nm TPBi thickness. With the optimized precursor NiO*_x_* solution (1.25 m), the current density, luminance, and device efficiency characteristics were measured for PeLEDs fabricated with different dimensionalities of perovskite (3D, *n* = 2, *n* = 3, and *n* = 5) deposited on NiO*_x_* and the optimized dimensionality (*n* = 3) of the perovskite deposited on PEDOT:PSS (**Figure** **4** and **Table** **1**). As the dimensionality of the perovskites decreased, the current densities of the devices gradually decreased because high fraction of 2D to 3D perovskite interrupted charge transport. The optimized dimensionality (*n* = 3) of the perovskite enabled efficient and fast energy transfer in the radiative domains by outpacing trapping and the subsequent nonradiative recombination, leading to improved luminance and device efficiency. Using the dimensionality (*n* = 3) of the perovskite, the device characteristics of PeLEDs fabricated with NiO*_x_* and PEDOT:PSS were compared. At low voltage, the PeLED fabricated with NiO*_x_* recorded lower leakage of current density than that with PEDOT:PSS (Figure 4a). Moreover, the PeLED fabricated with NiO*_x_* showed remarkably improved luminance (from 10 600 to 24 100 cd m^−2^), CE (from 17.4 to 62.4 cd A^−1^), and EQE (from 4.2% to 14.6%) compared with the PeLED fabricated with PEDOT:PSS. The obtained EQE of our green emissive device is 14.6%, which is comparable to the results reported so far (Table S3, Supporting Information). Such significantly improved device performance is attributed to the better interface formed at NiO*_x_*/perovskite layers with lower density of traps/defects, as well as more balanced charge carriers in the perovskite layer leading to high recombination yield of carriers.

**Figure 4:**
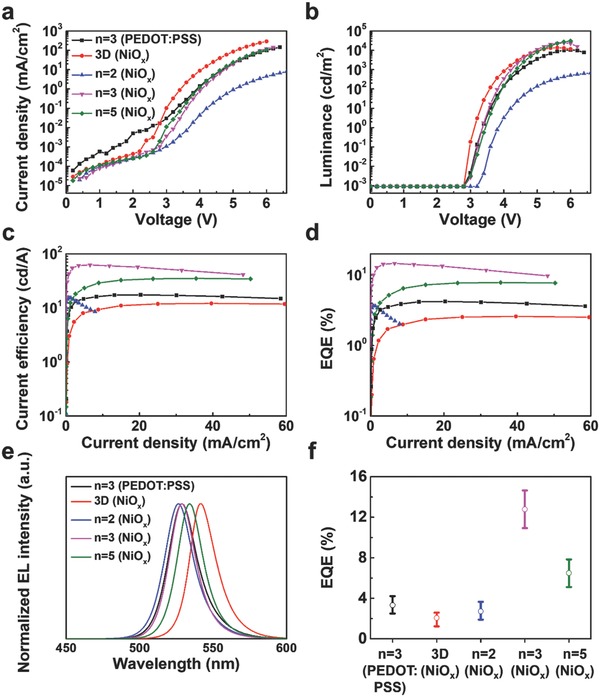
Device performance of PeLEDs fabricated with 3D FAPbBr_3_ and quasi‐2D perovskites with *n* = 2, 3, and 5 deposited on NiO*_x_* and quasi‐2D perovskite with *n* = 3 deposited on PEDOT:PSS. a) Current density versus voltage, b) luminance versus voltage, c) CE versus current density, d) EQE versus current density characteristics, e) normalized EL spectra, and f) EQE mean and deviation from each of 15 devices with 3D FAPbBr_3_ and quasi‐2D perovskites, with *n* = 2, 3, and 5 deposited on NiO*_x_* and quasi‐2D perovskite with *n* = 3 deposited on PEDOT:PSS.

**Table 1:** Summary of device performance of PeLEDs fabricated with 3D FAPbBr_3_ and quasi‐2D perovskites with *n* = 2, 3, and 5 deposited on NiO*_x_* and quasi‐2D perovskite with *n* = 3 deposited on PEDOT:PSS

Device configuration (PeLEDs)	Luminance_max_ [cd/m^2^]@bias	CE_max_ [cd A^−1^]@bias	EQE_max_ [%]@bias	EQE_avr_ [%] from 15 devices	Turn‐on voltage [V] @ 0.1 cd m^−2^
ITO/PEDOT:PSS/BA_2_FA_2_Pb_3_Br_10_ (*n* = 3)/TPBi/LiF/Al	10 600 @ 6.0 V	17.4 @ 5.0 V	4.22 @ 5.0 V	3.34	3.2
ITO/NiO*_x_*/FAPbBr_3_/TPBi/LiF/Al	13 700 @ 5.6 V	12.1 @ 4.6 V	2.59 @ 4.6 V	2.08	3.0
ITO/NiO*_x_*/BA_2_FAPb_2_Br_7_ (*n* = 2)/TPBi/LiF/Al	670 @ 6.6 V	15.5 @ 4.8 V	3.66 @ 4.8 V	2.74	3.6
ITO/NiO*_x_*/BA_2_FA_2_Pb_3_Br_10_ (*n* = 3)/TPBi/LiF/Al	24 100 @ 5.8 V	62.4 @ 4.6 V	14.64 @ 4.6 V	12.79	3.2
ITO/NiO*_x_*/BA_2_FA_4_Pb_5_Br_16_ (*n* = 5)/TPBi/LiF/Al	30 600 @ 6.0 V	35.1 @ 5.2 V	7.84 @ 5.2 V	6.50	3.2

The low interaction energy of perovskite allows many defects to easily form, leading to ion migration. The operational instability of PeLEDs may be due to the decomposition and interaction of perovskite with adjacent layers caused by ion migration. Defectless perovskite and adjacent layers can substantially improve operational stability. The operational stability of PeLEDs fabricated with quasi‐2D perovskite with *n* = 3 deposited on NiO*_x_* and PEDOT:PSS with encapsulation was measured at a luminance of 100 cd m^−2^ under ambient conditions as a function of operation time (**Figure** **5**a). The PeLED fabricated with NiO*_x_* exhibited remarkably improved operational stability compared with the PeLED fabricated with PEDOT:PSS. The PeLEDs fabricated with NiO*_x_* retained up to 90% and 50% of its initial luminance until 95 and 102 min, whereas the PeLED fabricated with PEDOT:PSS exhibited a sharp drop to less than 10% of the initial luminance within 8 min. Moreover, the electroluminescence (EL) spectra and CIE coordinates of PeLEDs fabricated with PEDOT:PSS and NiO*_x_* were measured to investigate the phase stability of quasi‐2D perovskite (Figure S11, Supporting Information). The EL spectra and CIE coordinates of both devices do not change over operating time, indicating the phase stability of FA‐based quasi‐2D perovskite. The instability of the PeLED fabricated with PEDOT:PSS may have been due to the acidic and hydroscopic nature of PEDOT:PSS as well as many interface defects between the perovskite and PEDOT:PSS. To investigate the effect of the defects in perovskite on device stability, EL microscope images of PeLEDs fabricated with NiO*_x_* and PEDOT:PSS were recorded over time (Figure 5b). The EL images of PeLED fabricated with NiO*_x_* showed bright emissions in all regions, where the brightness decreased over time. By contrast, the EL images of PeLED fabricated with PEDOT:PSS revealed large dark regions regarded as defects, and particularly brighter emissions near the dark regions. Brightness in boundaries of the dark regions slowly decreased compared with other regions over time. Regions of brightest near the defects showed a drastic reduction, forming the darkest regions. Although the exact mechanism of the phenomenon is challenging to probe and still under investigation, defects in the perovskite obviously impinge on the operational stability of PeLEDs.

**Figure 5:**
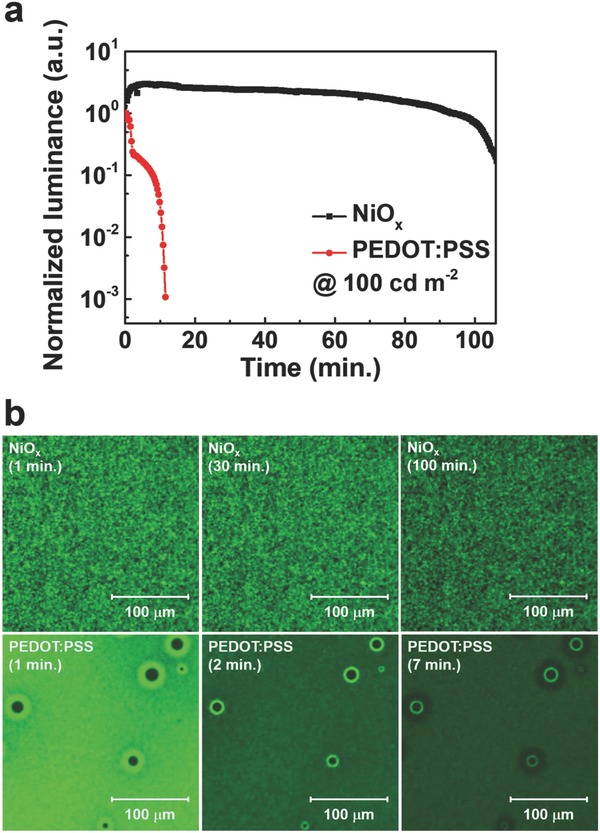
Operational stability and EL microscopic images of PeLEDs fabricated with quasi‐2D perovskite with *n* = 3 deposited on NiO*_x_* and PEDOT:PSS. a) Normalized luminance and b) EL microscope images of encapsulated PeLEDs fabricated with quasi‐2D perovskite with *n* = 3 deposited on NiO*_x_* and PEDOT:PSS as a function of operating time under ambient conditions.

In summary, we tested a high‐quality perovskite emitter with enhanced optical properties and thermal stability through compositional and dimensional modulations. FAPbBr_3_ showed a longer PL lifetime and higher PL intensity compared to MAPbBr_3_, owing to its low trap density. The dimensional modulation of FA‐based perovskite enabled long‐living free carriers to recombine in small radiative domains through efficient and fast energy transfer, which substantially improved the PL intensity with improvement of PLQY to 53.1%. Moreover, we investigated the energetics and trap density of states at the interface between the perovskite and HTL (NiO*_x_* and PEDOT:PSS). The NiO*_x_* film allowed the growth of highly crystalline perovskite with reduced trap density of states compared with PEDOT:PSS, which led to enhanced optical properties, the photostability of perovskite, and the operational stability of the PeLEDs. With effective control over the trap density of perovskite, efficient and stable green emissive PeLEDs with a maximum luminance of 24 100 cd m^−2^, a maximum CE of 62.4 cd A^−1^, and a maximum EQE of 14.6% were achieved.

## Experimental Section

*Materials*: PEDOT:PSS (AI 4083, Clevios) and nickel acetate tetrahydrate (99.998%, Sigma Aldrich) were used without being subjected to any purification. PbBr_2_ (99.999%, Alfa Aesar), FABr (Dyesol), BABr (98%, Tokyo Chemical Industry), and TPBi (99.9%, OSM) were used without further purification.

*Device Fabrication*: A patterned ITO/glass substrate was cleaned using an ultrasonic bath in deionized water, acetone, and isopropanol for 10 min. PEDOT:PSS dispersion was spin‐coated at 5000 rpm for 40 s onto the ultraviolet‐ozone treated ITO substrate, and was then annealed at 140 °C for 10 min at in a nitrogen‐filled glove box. To prepare NiO*_x_* precursor, nickel acetate tetrahydrate with ethanolamine (mole ratio of 1:1) was dissolved in 2‐methoxyethanol string 80 °C for 4 h. The precursor was spin‐coated at 4000 rpm for 45 s and annealed at 500 °C for 1 h in ambient conditions. To prepare the precursors, PbBr_2_, FABr, and BABr at a molar ratio of 1:1:0 (3D), 1:0.50:1 (*n* = 2), 1:0.67:0.67 (*n* = 3), and 1:0.80:0.40 (*n* = 5) were dissolved in a dimethylformamide/dimethyl sulfoxide (7:3 v/v) co‐solvent (0.3 m) at 60 °C. The perovskite precursor was first spin‐coated onto the substrate at 3000 rpm for 80 s on the HTL using a hydrophilic filter (pore size 0.45 µm). After a delay of 30 s, 80 µL of the chlorobenzene antisolvent solution was dropped and spin‐cast onto the precursor film in the nitrogen‐filled glove box. TPBi as electron transport layers (60 nm) were deposited using a thermal evaporation system at an evaporation rate of 0.5 Å s^−1^. Finally, for electrode formation, LiF (1 nm) and Al (100 nm) were successively deposited using a thermal evaporation system at the high vacuum of ≈1 × 10^−6^ Torr.

*Device Characterization*: The *J–V–L* and device efficiencies of the PeLEDs with encapsulation were obtained using a computer‐controlled Keithley 2400 Source Meter and a Konica Minolta spectroradiometer (CS‐2000, Minolta) under ambient conditions.

*Time‐Resolved and Steady‐State PL Measurements*: Time‐resolved and steady‐state PL spectra were measured using a time‐correlated single‐photon counting (TCSPC) setup (FluoTime 300), described elsewhere.^3^ The excitation source was a 450 nm continuous wave and pulsed diode laser head (LDH‐D‐C‐450) coupled with a laser diode driver (PDL 820) with a pulse width of <70 ps and a repetition rate of 196 kHz–40 MHz. The time‐resolved PL signal was obtained using a TCSPC module (PicoHarp) with a photomultiplier tube (PMA‐C 182‐N‐M). Each exponential decay curve was deconvoluted using the associated fitting software (FluoFit) to calculate the time constant associated with each curve.

*SEM Measurements*: SEM measurements were performed using a Nanonova 230 FEI SEM with an accelerating voltage of 10 kV. A 5 nm platinum layer was deposited on each perovskite film to prevent any charging effects using a sputter coater (Emitech K575x, Tescan).

*XRD Measurements*: XRD measurements were performed using a D/MAX2500V/PC (Rigaku, Japan) equipped with a Cu Kα radiation source (λ = 1.5405 Å). The step size was 0.02°, with an acquisition time of 60 s deg^−1^.

*PLQY Measurement*: The PLQY of the perovskite films were obtained using an integrating sphere method. The samples were excited using a 407 nm continuous wave diode laser with a focused beam spot of ≈0.3 mm^2^ and an excitation intensity of 48 mW. An Andor iDus DU490A InGaAs detector was used to measure emission. The details have been described elsewhere.^1^

*Confocal Fluorescence Imaging*: Confocal PL images of the perovskite films were measured using an LSM 780 NLO laser scanning confocal microscope (Carl Zeiss) with a 100× oil immersion objective (a Plan‐APO, NA = 1.46). The excitation source was a 405 nm diode laser.

*EL Microscopy*: EL microscopy images of the PeLED samples fabricated with quasi‐2D perovskite film with *n* = 3 deposited on NiO*_x_* and PEDOT:PSS were obtained using an inverted microscope (IX81, Olympus).

*Measurements of APS, KP, and SPV*: Measurements of APS, KP, and SPV were made using the KP technology APS‐04 instrument. For APS measurements, the sample was illuminated with UV light from a monochromatic deuterium lamp source (4–7 eV). The raw photoemission data were corrected for offset and the cube root was taken, with the HOMO value found from the extrapolated intersection of the straight‐line fit of the cube root of the photoemission with the baseline.

KP WF measurements were taken by using a 2 mm gold alloy‐coated vibrating tip above the surface of the sample, with the resulting contact potential difference between the tip and the sample added to the WF of the gold tip to find the resultant WF of the sample. For SPV measurements, the sample was illuminated with a 150 W quartz halogen lamp coupled by fiber optic and focused on the sample.

## Conflict of Interest

The authors declare no conflict of interest.

## Supporting information

Supplementary
