# Supplementary material for: Control of Interface Defects for Efficient and Stable Quasi‐2D Perovskite Light‐Emitting Diodes Using Nickel Oxide Hole Injection Layer
Source: Adv Sci (Weinh). 2018 Oct 4;5(11):1801350. doi: 10.1002/advs.201801350 (PMC6247068; doi:10.1002/advs.201801350)
Supplement: Supplementary file 1 — Supplementary [file ADVS-5-1801350-s001.pdf]

Copyright WILEY-VCH Verlag GmbH & Co. KGaA, 69469 Weinheim, Germany, 2018.

ADVANCED SCIENCE Open Access

Supporting Information

for *Adv. Sci.,* DOI: 10.1002/advs.201801350

Control of Interface Defects for Efficient and Stable Quasi-2D Perovskite Light-Emitting Diodes Using Nickel Oxide Hole Injection Layer

*Seungjin Lee, Da Bin Kim, Iain Hamilton, Matyas Daboczi, Yun Seok Nam, Bo Ram Lee, Baodan Zhao, Chung Hyeon Jang, Richard H. Friend, Ji-Seon Kim, and Myoung Hoon Song**

WILEY-VCH

Copyright WILEY-VCH Verlag GmbH & Co. KGaA, 69469 Weinheim, Germany, 2016.

Supporting Information

Interfacial Control for Efficient and Stable Quasi-2D Perovskite Light-Emitting Diodes Using Nickel Oxide Hole Injection Layer

*Seungjin Lee, Da Bin Kim, Iain Hamilton, Matyas Daboczi, Yun Seok Nam, Bo Ram Lee, Baodan Zhao, Chung Hyeon Jang, Richard H. Friend, Ji-Seon Kim, Myoung Hoon Song**

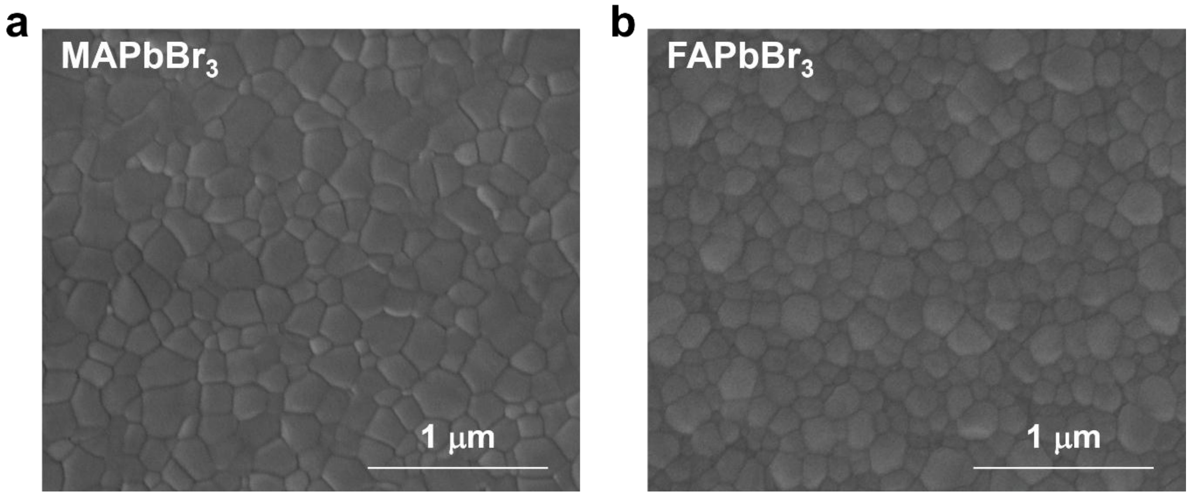

Figure S1. SEM images of $FAPbBr_3$ and $MAPbBr_3$. SEM images of a) 3D $MAPbBr_3$ and b) 3D $FAPbBr_3$.

$FAPbBr_3$ and $MAPbBr_3$ have similar morphologies and grain sizes obtained through the same fabrication method and conditions. The SEM images of $FAPbBr_3$ and $MAPbBr_3$ showed full coverage and smooth morphologies without any pinhole.

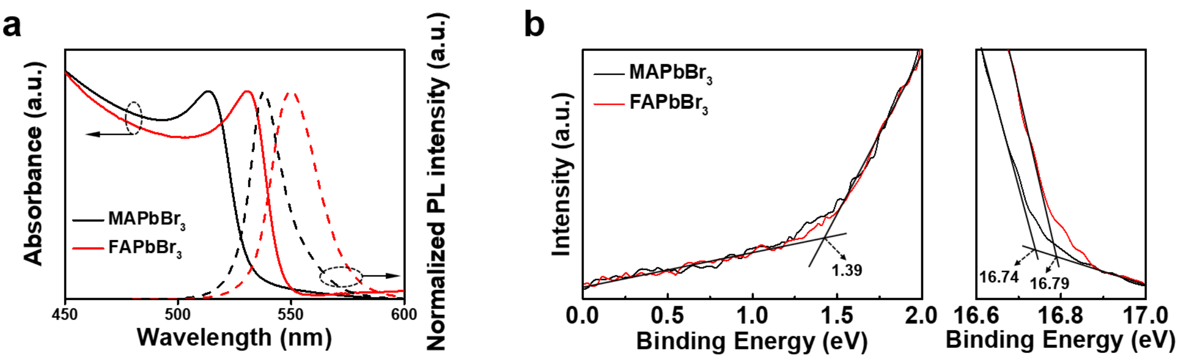

Figure S2. Absorbance and UPS of $MAPbBr_3$ and $FAPbBr_3$. a) Absorbance and normalized PL spectra of $MAPbBr_3$ and $FAPbBr_3$. b) UPS data of $MAPbBr_3$ and $FAPbBr_3$.

$FAPbBr_3$ showed red-shifted band edge absorption and PL emission owing to a smaller band gap than $MAPbBr_3$. To investigate the energy levels of the conduction band and valence band of $MAPbBr_3$ and $FAPbBr_3$, we measured absorbance and UPS. We measured the optical band gaps of $MAPbBr_3$ and $FAPbBr_3$ at 2.34 and 2.27 eV, respectively, and the valence bands of $MAPbBr_3$ and $FAPbBr_3$ at 5.87 and 5.82 eV, respectively.

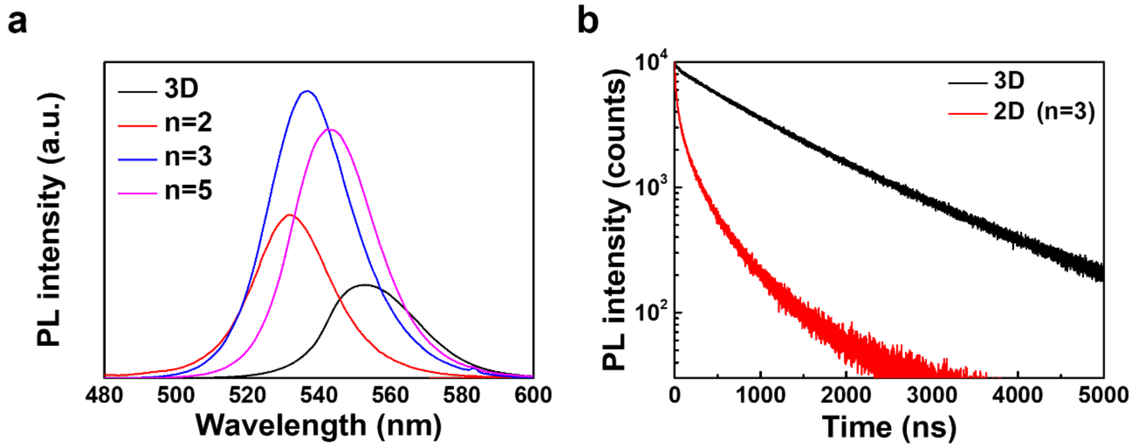

Figure S3. Optical properties of 3D $FAPbBr_3$ and quasi-2D perovskite. a) Steady-state PL spectra of 3D $FAPbBr_3$ and quasi-2D perovskite with n = 2, n = 3, and n = 5. b) Time-resolved PL spectra of 3D $FAPbBr_3$ and quasi-2D perovskite with n = 3.

Dimensional modulation concentrates carriers on a smaller band gap emitter through energy transfer, which increases the bimolecular radiative recombination by outcompeting the trap-mediated non-radiative recombination. The PL intensities of the quasi-2D perovskite are higher than that of 3D $FAPbBr_3$, and among them, quasi-2D perovskite with n = 3 shows the highest PL intensity through efficient and fast energy transfer. Quasi-2D perovskite with n = 3 shows a substantially shorter PL lifetime (0.14 μs) than 3D $FAPbBr_3$ (4.66 μs), which indicates that dimensional modulation enables long-living free carriers to recombine in small radiative domains by effectively concentrating them in small radiative domains.

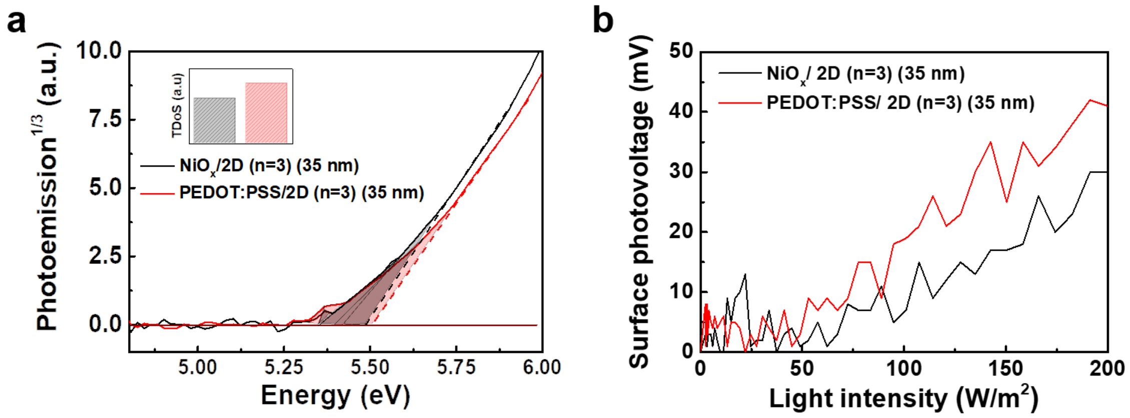

Figure S4. APS and SPV measurements for quasi-2D perovskite (35 nm) with n = 3 deposited on NiO_x and PEDOT:PSS. a) APS spectra for 35-nm thick perovskite films deposited on NiO_x and PEDOT:PSS. b) SPV for 35-nm thick perovskite films deposited on NiO_x and PEDOT:PSS.

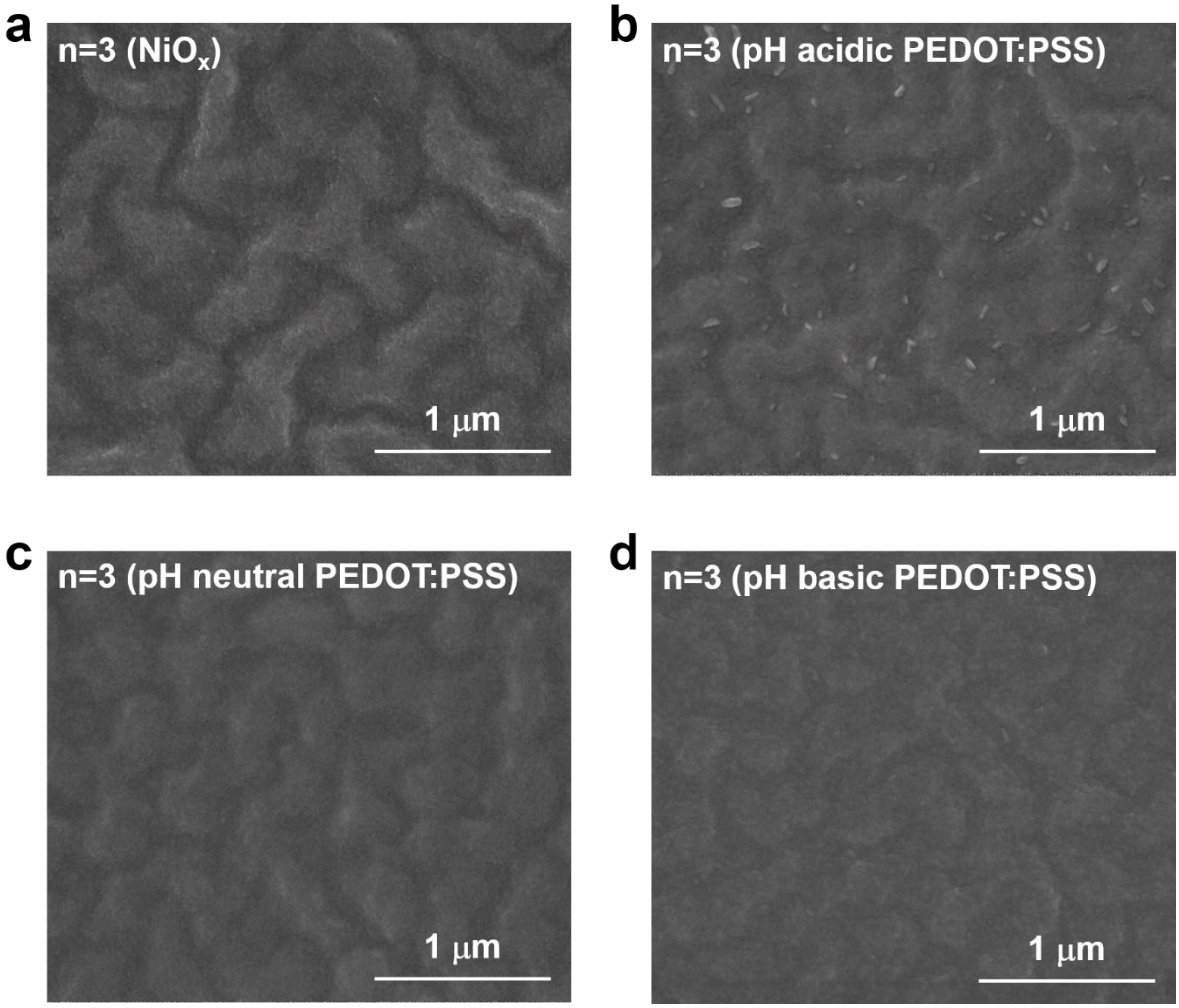

Figure S5. SEM images of perovskite films on NiOx and PEDOT:PSS with different pH values. SEM images of quasi-2D perovskite with n = 3 deposited on a) NiO_x, b) pH acidic, c) pH neutral, and d) pH basic PEDOT:PSS.

The crystalline surface can assist the perovskite nucleation process during film formation. The crystalline NiO_x film can enable the growth of highly crystalline perovskite and reduce trap states at the interface of the perovskite and HTL. We measured the morphologies of the perovskite films deposited on NiO_x and PEDOT:PSS. The surface of the perovskite film deposited on NiO_x showed neat morphology without no other shape of crystallite. In contrast, the surface of the perovskite film deposited on PEDOT:PSS was intricately covered with various shapes of crystallites, which may be attributed to the acidic characteristics of PEDOT:PSS. The morphologies of the perovskite films deposited on PEDOT:PSS with different pH values were compared to investigate the effect of its acidity on the growth of perovskite film. PEDOT:PSS (AI 4083, Clevios) was titrated with imidazole to adjust pH values of PEDOT:PSS. The pH values of acidic, neutral and basic PEDOT:PSS were measured to be 1.95, 7.5 and 8.5 using pH meter. The surface of the perovskite films

deposited on pH neutral and basic PEDOT:PSS showed clean morphology without no crystallite, which indicates that the acidic characteristics of PEDOT:PSS have a negative effect on crystal growth.

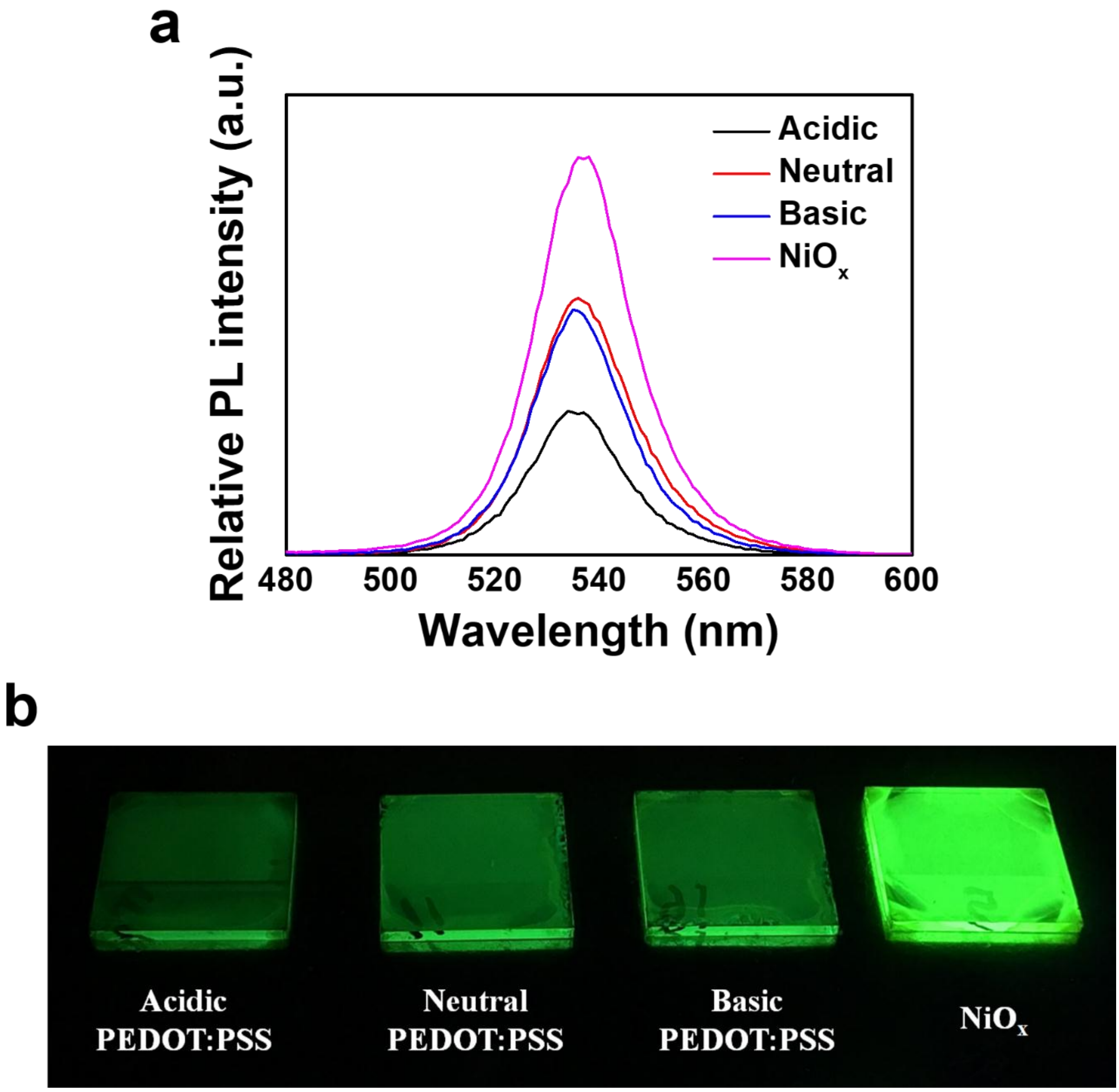

Figure S6. Optical properties of perovskite films deposited on NiO_x and PEDOT:PSS with different pH values. a) Steady-state PL spectra and b) photographs showing the green PL emission of perovskite films deposited on NiO_x and PEDOT:PSS with different pH values.

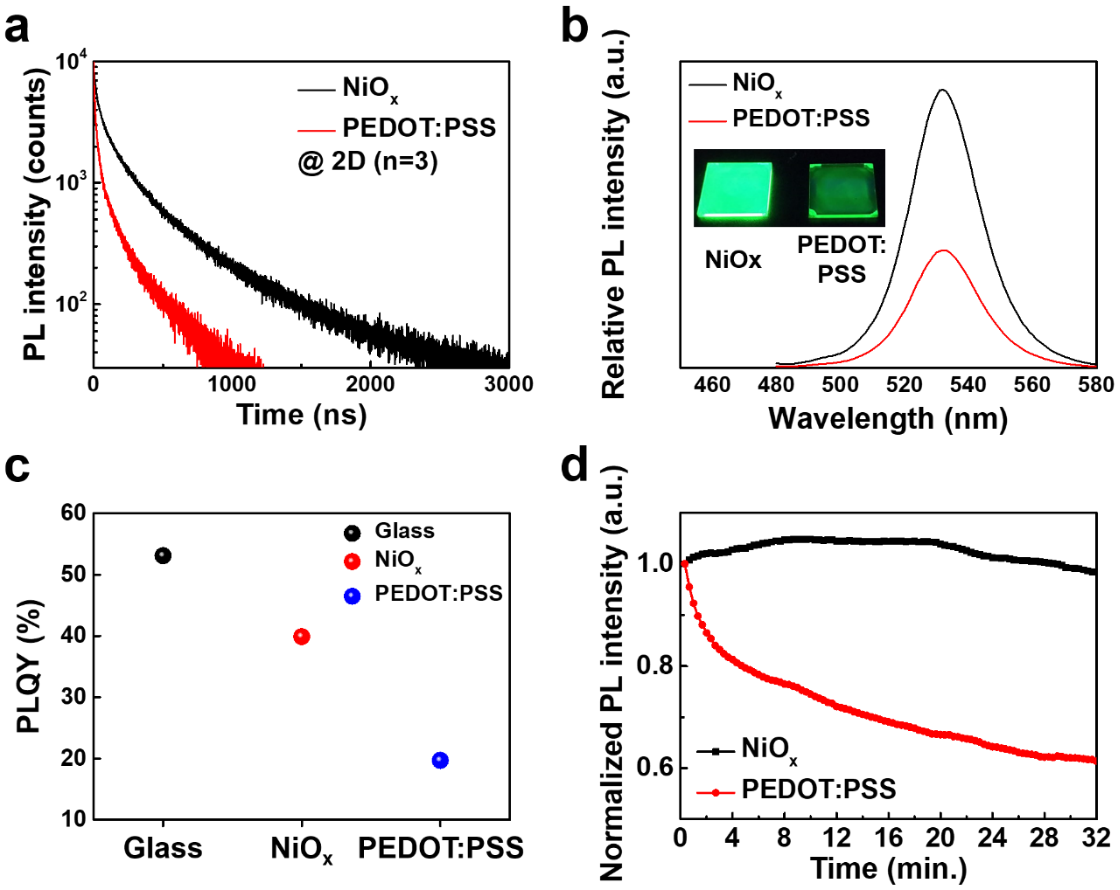

Figure S7. Optical properties and stability of perovskite films deposited on NiO_x and PEDOT:PSS. a) Time-resolved PL spectra, and b) steady-state PL spectra of quasi-2D perovskite with n = 3 deposited on NiO_x and PEDOT:PSS. c) PLQYs of quasi-2D perovskite with n = 3 deposited on glass, NiO_x, and PEDOT:PSS. d) Normalized PL intensity of quasi-2D perovskite with n = 3 deposited on NiO_x and PEDOT:PSS during excitation by a 405-nm laser as a function of time.

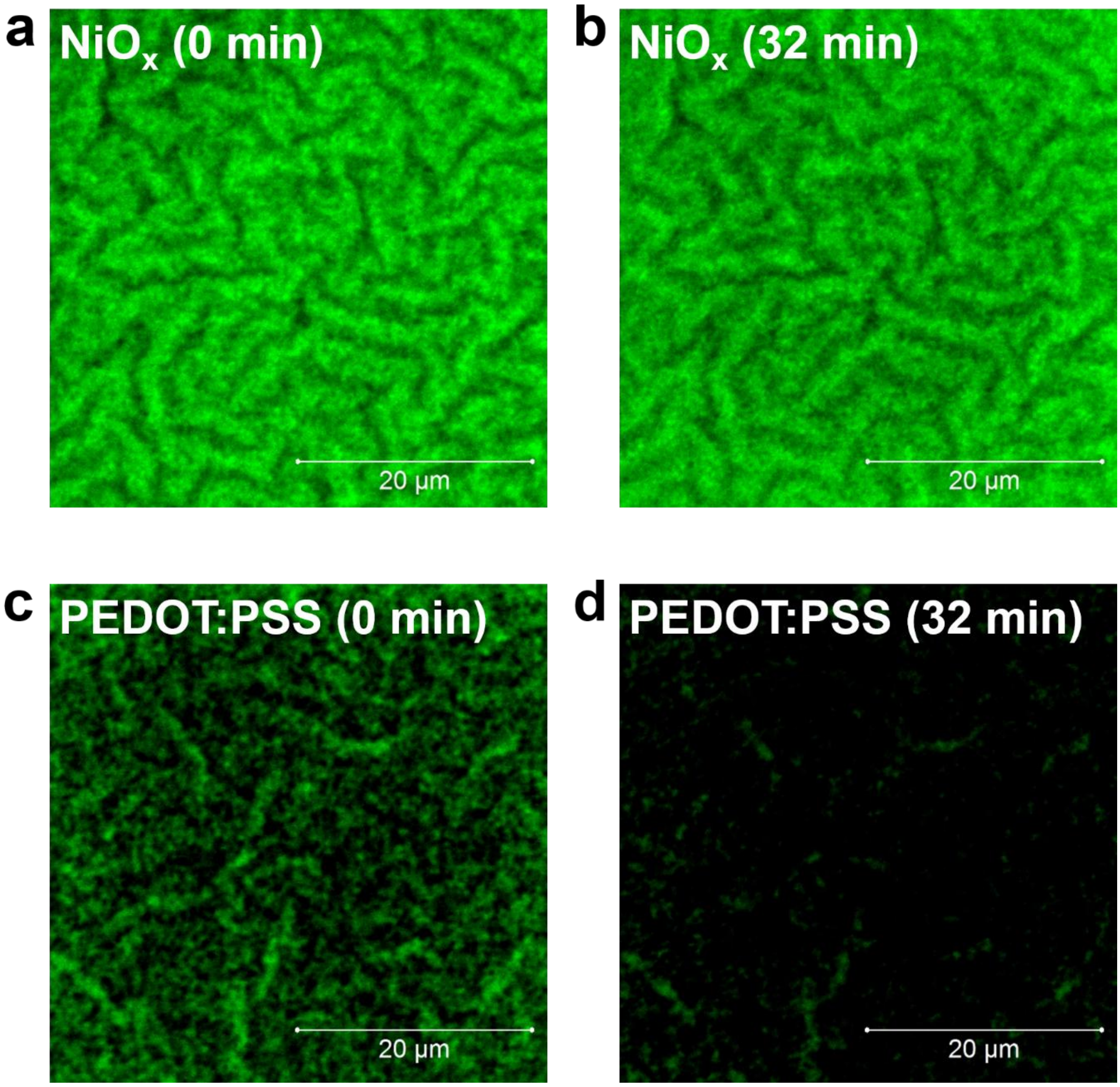

Figure S8. Optical Stability of perovskite films on NiO_x and PEDOT:PSS (confocal microscopy). Confocal PL images of quasi-2D perovskite with n = 3 deposited on NiO_x with excitation by a 405-nm laser after a) 0 min, and b) 32 min. Confocal PL images of quasi-2D perovskite with n = 3 deposited on PEDOT:PSS with excitation by a 405-nm laser excitation after c) 0 min, and d) 32 min.

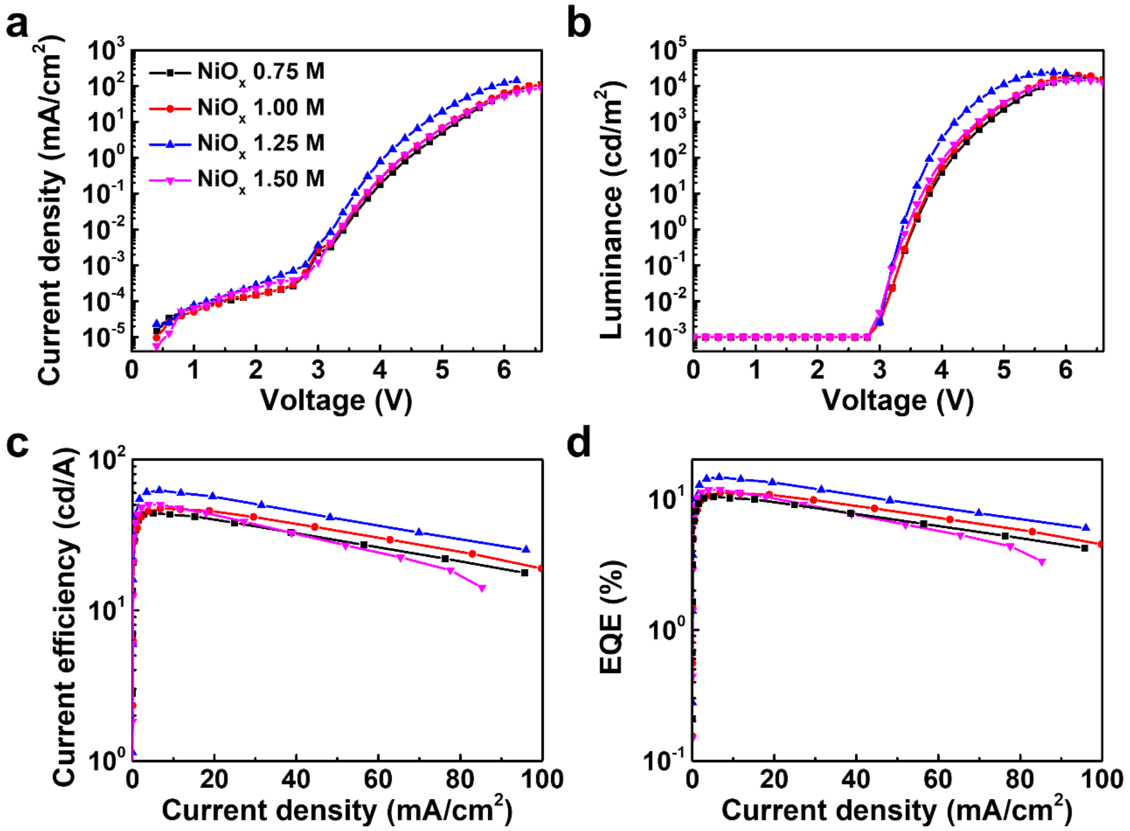

Figure S9. Device performance of PeLEDs fabricated with quasi-2D perovskite with n = 3 and different concentrations of NiO_x precursor. a) Current density versus voltage, b) luminance versus voltage, c) CE versus current density, and d) EQE versus current density of the PeLEDs fabricated with quasi-2D perovskite with n = 3 and different concentrations of NiO_x precursor.

The NiO_x films were optimized by testing different concentrations of the precursor NiO_x solution to balance the charge carriers through efficient hole transport and electron blocking. The performance of the device changed with the thickness of NiO_x. PeLEDs with an optimal concentration of the NiO_x precursor (1.25 M) exhibited the maximum luminance and efficiency, with balanced charge carriers in the perovskite layer.

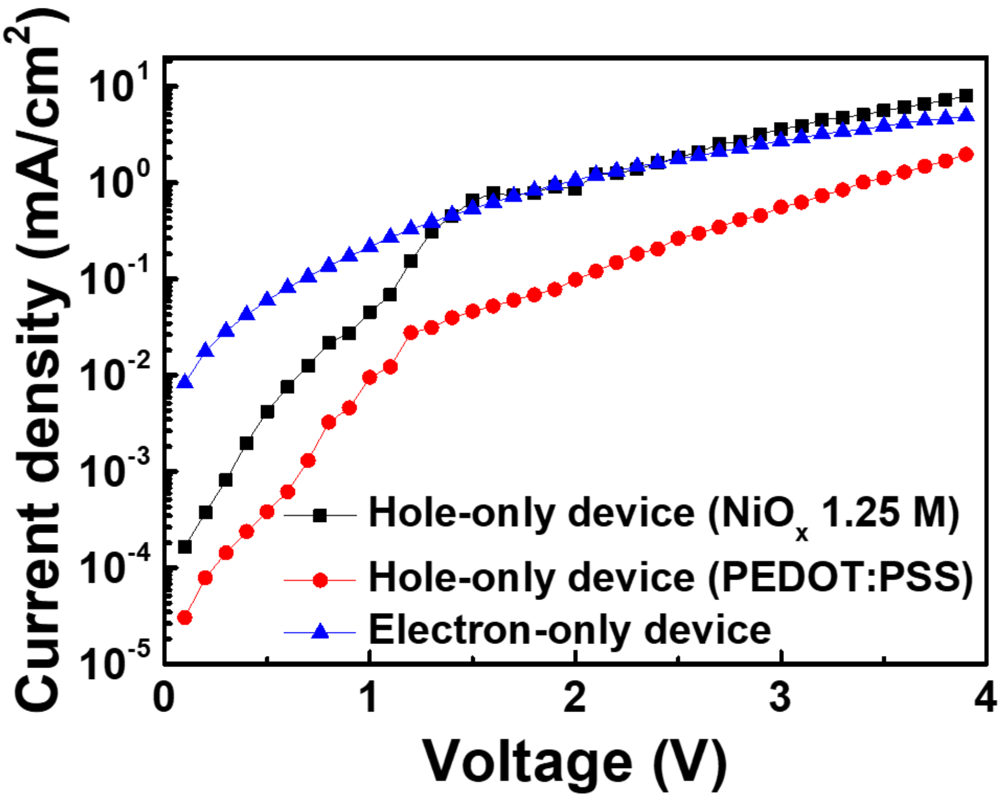

Figure S10. *J*-*V* characteristics of hole-only devices (ITO/NiO_x or PEDOT:PSS/$BA_2FA_2Pb_3Br_{10}$/TFB/MoO_3/Au) and electron-only device (ITO/ZnO/ $BA_2FA_2Pb_3Br_{10}$/TPBi (60 nm)/LiF/Al).

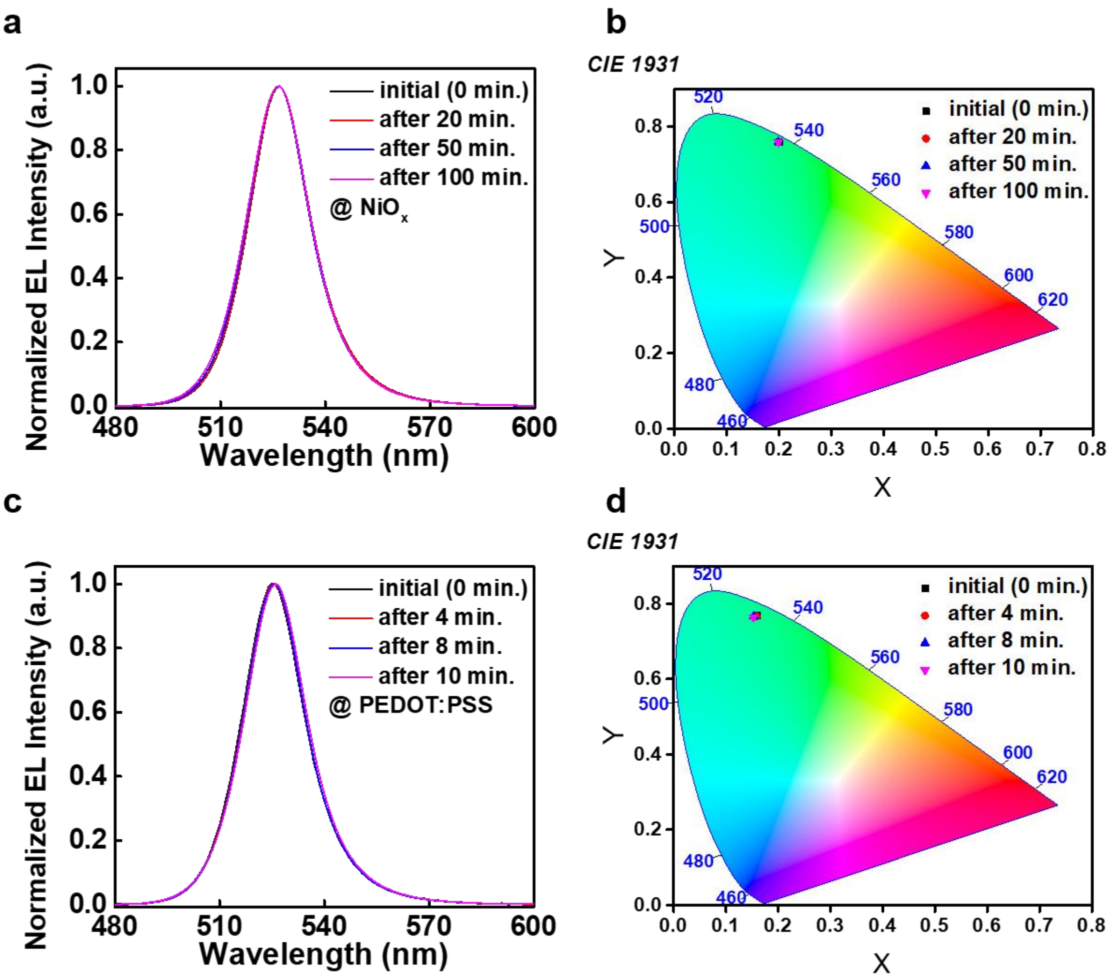

Figure S11. EL spectral stability of PeLEDs fabricated with NiO_x as a function of operating time under ambient conditions. a) Normalized EL spectra and b) CIE coordinates of PeLEDs fabricated with NiO_x as over operating time. c) Normalized EL spectra and d) CIE coordinates of PeLEDs fabricated with PEDOT:PSS as over operating time.

Table S1. Summarized PL lifetimes of perovskite films deposited on NiO_x and PEDOT:PSS.

**Film configuration**	**τ_{avr} [μs]**	**χ^2**
Glass / NiO_x / 3D $MAPbBr_3$	0.01	1.2711
Glass / NiO_x / 3D $FAPbBr_3$	4.66	1.2711
Glass / NiO_x / $BA_2FA_2Pb_2Br_{10}$ (n=3)	0.14	1.2338
Glass / PEDOT:PSS / $BA_2FA_2Pb_2Br_{10}$ (n=3)	0.04	1.3697

Table S2. Summary of device performance of PeLEDs fabricated with quasi-2D perovskite with n = 3 and different concentrations of the NiO_x precursor.

**Device configuration (PeLEDs)**	**L_{max} [cd/m²] @ bias**	**CE_{max} [cd/A] @ bias**	**EQE_{max} [%] @ bias**	**Turn-on voltage [V] @ 0.1 cd/m²**
ITO / NiO_x (0.75 M) / $BA_2FA_2Pb_3Br_{10}$ / TPBi / LiF / Al	16,900 @ 6.4 V	44.1 @ 5.0 V	10.4 @ 5.0 V	3.2
ITO / NiO_x (1.00 M) / $BA_2FA_2Pb_3Br_{10}$ / TPBi / LiF / Al	19,600 @ 6.2 V	47.3 @ 5.0 V	11.1 @ 5.0 V	3.2
ITO / NiO_x (1.25 M) / $BA_2FA_2Pb_3Br_{10}$ / TPBi / LiF / Al	24,100 @ 5.8 V	62.4 @ 4.6 V	14.6 @ 4.6 V	3.2
ITO / NiO_x (1.50 M) / $BA_2FA_2Pb_3Br_{10}$ / TPBi / LiF / Al	14,700 @ 6.2 V	50.2 @ 5.0 V	11.8 @ 5.0 V	3.2

Table S3. Comparison of our work with previous reports.

**Publication year/month**	**Device structure**	**Emission wavelength**	**Max. EQE (%)**	**Max. CE (cd/A)**	**Max. L (cd/m^2)**	**Device Stability**	**Ref.**
	$ITO/NiO_x/BA_2FA_2Pb_3Br_{10}/TPBi/LiF/Al$	543nm	14.64	62.4	24,100	L_{90}≈95 min. L50≈102 min.	**Our work**
2015/12	$PEDOT:PSS:PFI/CH_3NH_3PbBr_3/TPBi/LiF/Al$	543nm	8.53	45.9	~20,000	-	[1]
2017/01	$ITO/PVK/BA(MAPbBr_3)_2PbBr_4/TPBi/LIF/Al$	513nm	9.3	17.1	2,900	L_{50} ≈ 48min.	[2]
2017/03	$ITO/PEDOT:PSS/MAPbBr_3/TPBi/LiF/Al$	540nm	8.21	34.46	6,950	L_{50} ≈ 100 sec.	[3]
2017/04	$ITO/PEDOT:PSS/MAPbBr_3/EDA/$ SPW-111/LiF/Al	540nm	6.2	28.9	22,800	L_{70} ≈ 4h.	[4]
2017/06	$ITO/ZnO/PVP/Cs_{0.87}MA_{0.13}PbBr_3/CBP/MoO_3/Al$	520nm	10.43	33.9	91,000	L_{50} ≈ 60min.	[5]
2018/02	$ITO/mPEDOT:PSS/PEA_2(FAPbBr_3)_2PbBr_4/TPBi/LiF/Al$	532nm	14.36	62.43	9,120	L_{50} ≈ 60min.	[6]
2018/05	$ITO/PEDOT:PSS/MAPbBr_3/TPBi/LiF/Al$	538nm	12.1	55.2	55,400	L_{50} ≈ 60min.	[7]

Reference for Supporting Information

[1] H. C. Cho, S. H. Jeong, M. H. Park, Y. H. Kim, C. Wolf, C. L. Lee, J. H. Heo, A. Sadhanala, N. Myoung, S. Yoo, S. H. Im, R. H. Friend, T. W. Lee, *Science* **2015**, *350*, 1222.

[2] Z. G. Xiao, R. A. Kerner, L. F. Zhao, N. L. Tran, K. M. Lee, T. W. Koh, G. D. Scholes, B. P. Rand, *Nat. Photon.* **2017**, *11*, 108.

[3] J. W. Lee, Y. J. Choi, J. M. Yang, S. Ham, S. K. Jeon, J. Y. Lee, Y. H. Song, E. K. Ji, D. H. Yoon, S. Seo, H. Shin, G. S. Han, H. S. Jung, D. Kim, N. G. Park, *ACS Nano* **2017**, *11*, 3311.

[4] S. J. Lee, J. H. Park, B. R. Lee, E. D. Jung, J. C. Yu, D. Di Nuzzo, R. H. Friend, M. H. Song, *J. Phys. Chem. Lett.* **2017**, *8*, 1784.

[5] L. Q. Zhang, X. L. Yang, Q. Jiang, P. Y. Wang, Z. G. Yin, X. W. Zhang, H. R. Tan, Y. Yang, M. Y. Wei, B. R. Sutherland, E. H. Sargent, J. B. You, *Nat. Commun.* **2017**, *8*, 15640.

[6] X. L. Yang, X. W. Zhang, J. X. Deng, Z. M. Chu, Q. Jiang, J. H. Meng, P. Y. Wang, L. Q. Zhang, Z. G. Yin, J. B. You, *Nat. Commun.* **2018**, *9*, 570.

[7] S. Lee, J. H. Park, Y. S. Nam, B. R. Lee, B. D. Zhao, D. Di Nuzzo, E. D. Jung, H. Jeon, J. Y. Kim, H. Y. Jeong, R. H. Friend, M. H. Song, *ACS Nano* **2018**, *12*, 3417.
